# Risk Factors for Postoperative Unplanned Reintubation in a Cohort of Patients Undergoing General Anesthesia

**DOI:** 10.7759/cureus.38949

**Published:** 2023-05-12

**Authors:** Iwan Sofjan, Sima Vazquez, Jose Dominguez, Nitin Sekhri, Matthew Wecksell, Barst M Samuel, Irim Salik

**Affiliations:** 1 Anesthesiology, Westchester Medical Center, Valhalla, USA; 2 Medicine, New York Medical College, Valhalla, USA; 3 Neurosurgery, Westchester Medical Center, Valhalla, USA

**Keywords:** risk factors, opioids, perioperative complications, unplanned reintubation, reintubation

## Abstract

Background

Unplanned post-operative reintubation (UPR) is a complication of general anesthesia (GA) that can be associated with worsened outcomes.

Objective

Evaluate characteristics associated with UPR in patients undergoing procedures under GA.

Methods

Patients over the age of 18 undergoing surgical procedures under GA were extracted from our institution's electronic medical record. Patient baseline, procedural, and anesthesia characteristics were evaluated for associations with UPR.

Results

In 29,284 surgical procedures undergoing GA, there were 29 (0.1%) patients that required UPR. The most common surgical service with UPR was otolaryngology; the most common surgical positioning was supine. When controlling for operative time and case complexity, UPR was predicted by high-dose opioids, defined as opioid administration greater than the 75th percentile of our institutional cohort. Prolonged operative time, estimated blood loss (EBL), body mass index (BMI), extubation time after reversal, or age were not independently associated with UPR.

Conclusion

Our analysis revealed that high-dose opioid administration is independently associated with intraoperative UPR. Awareness of patients at the highest risk for UPR along with provider education regarding techniques to avoid respiratory depression in this patient population is essential in reducing patient morbidity and mortality. This knowledge will help guide perioperative physicians in medical optimization, appropriate selection of intraoperative analgesics, and cautious extubation criteria to ensure patient safety.

## Introduction

Unplanned post-operative reintubation (UPR) is an anesthesia complication associated with unfavorable outcomes across surgical patient populations [1-3]. Although it can be a life saving measure for the patient in extremis, it remains a harbinger for an unfavorable postoperative course. Risk factors for UPR include certain patient, procedural, and anesthetic characteristics [4,5] Post-operative complications such as UPR can have a significant negative impact on patient outcomes, quality metrics, and hospital reimbursement [6,7]. Further elucidation of underlying risk factors may aid in extubation planning and maintaining vigilance for high-risk patients at risk of post-operative airway compromise. Because UPR is a relatively rare perioperative complication with an incidence between 0.14 - 0.19%, we opted to use a large cohort of patients at our institution to evaluate risk factors in patients undergoing general anesthesia [8].

## Materials and methods

Data source and patient selection

This study was approved by our hospital’s Institutional Review Board. The electronic medical record was queried for patients undergoing procedures under general anesthesia during the time frame of October 10, 2020, to October 10, 2022, was included. Patients undergoing postoperative re-intubation over the age of 18 were identified and included for analysis of demographics, and surgical and anesthetic-specific factors. 

Data characteristics and outcomes measured

Patient demographics, including age, race, and ethnicity were described. American Society of Anesthesiologists (ASA) physical status and body mass index (BMI) were recorded. Case controls were matched based on age and procedure type. Characteristics of the operation, such as the experience level of an anesthesiologist, surgical service, operative time, narcotics, blood products, crystalloid, colloid, estimated blood loss (EBL), and patient positioning were analyzed. Indications of re-intubation were also described. High-dose opioid was defined as morphine or fentanyl administered above the 75th percentile of the cohort. Prolonged extubation time after reversal, increased EBL, and increased BMI were defined as time, blood loss, and weight greater than the 75th percentile of the patient cohort.

Statistical analysis

Categorical variables were compared using Pearson’s chi-squared test. Continuous variables were evaluated for normality using the Kolmogorov-Smirnov Test. Normally and non-normally distributed continuous variables were tested using the Student’s t-test and Mann-Whitney U test, respectively. Multivariate regression analysis was utilized to evaluate predictors of reintubation. Statistical Product and Service Solutions (SPSS) Statistical Software (IBM Corp. Released 2020. IBM SPSS Statistics for Windows, Version 28.0. Armonk, NY: IBM Corp) was used for analysis, and statistical significance was set at < 0.05.

## Results

General characteristics 

Of the 29,284 general anesthesia cases, there were 29 (0.1%) patients that underwent UPR. There were 19 (65.5%) Caucasian, 4 (13.8%) Black/African American, and 4 (13.8%) Hispanic. The median BMI was 23.00 (IQR 18.00-31.50). The median time to extubation after reversal was 14.50 minutes (IQR 5.00-25.25). Ten of these (34.5%) patients who underwent UPR were obese (BMI > 30). ASA class 3 (10, 34.5%) was the most common, followed by ASA 2 (7, 24.1%) and ASA 3E (5, 17.2%). Three (10.3%) patients were ASA 1, two (6.9%) were ASA 4, and two (6.9%) were ASA 4E.

Intraoperative characteristics 

**Figure 1:**
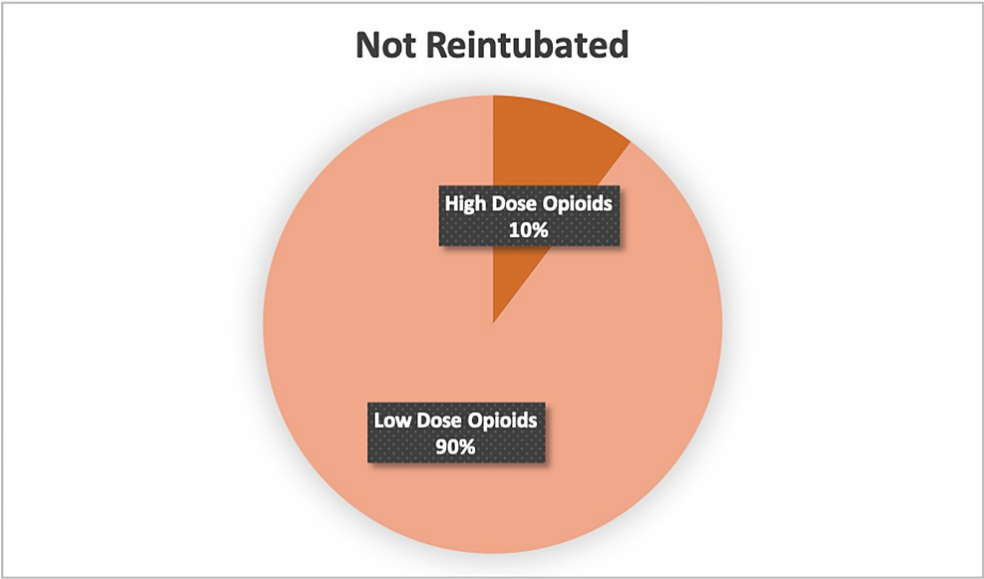
Percentage of Non-Reintubated Patients who Received High Dose Opioids Pie graph depicting distribution of high opioids amongst patients who were not reintubated.

**Figure 2:**
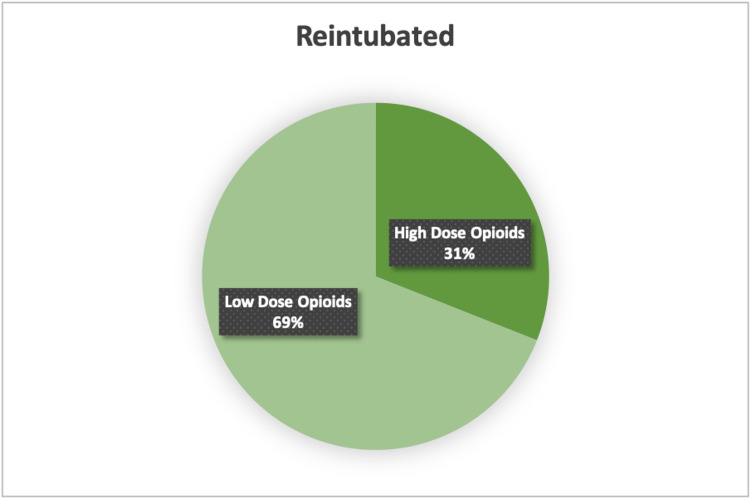
Percentage of Reintubated Patients who Received High Dose Opioids Pie graph depicting distribution of high dose opioids amongst patients who were reintubated.

UPR patients had a median operative time of 133 minutes (IQR 43-178.50). Vasopressors were required in four (13.8%), colloid was given in five (17.2%), and the median crystalloid administered during the operation was 800 mL (IQR 400-1250). Four (13.8%) patients received intraoperative hydromorphone. The median fentanyl administered was 175 mcg (IQR 45-250) and the median morphine administered was 17.50 mg (IQR 4.50-25.0). Five (17.2%) patients who underwent reintubation had received blood products intra-operatively, and the median estimated blood loss (EBL) was 8 mL (0-100). High-dose opioids were administered to 31% of the patients who were reintubated (Figures 1, 2).

Most patients underwent surgery in the supine position (20, 69.0%). The otolaryngology service was the most represented, accounting for 31.0% (n=9) of patients who underwent UPR. This was followed by general surgery (5, 17.2%), neurosurgery (5, 17.2%), and dentistry (3, 10.3%).

Indications for intubation

The most common indication for reintubation was poor respiratory effort (6, 20.7%). Five (17.2%) patients underwent UPR for laryngospasm or bronchospasm, and five (17.2%) underwent UPR for unknown reasons. Four (13.8%) patients underwent UPR for bleeding; three (10.3%) for mucus plug, cuff leak, or unintended extubation each. The operating room was the setting for 28 (96.6%) of UPR (Table 1).

**Table 1:** Characteristics of Patients who underwent Reintubation Descriptive statistics depicting baseline demographics, intubation characteristics, operation characteristics, and characteristics of patients who underwent reintubation.

	n=29
Demographics	
White	19 (65.5%)
Black/African American	4 (13.8%)
Hispanic	4 (13.8%)
Other	2 (6.9%)
BMI (Median (IQR))	23.00 (18.00-31.50)
BMI > 29	10 (34.5%)
Age (Median (IQR))	51 (4-62)
Intubation Characteristics	
ASA 1	3 (10.3%)
ASA 2	7 (24.1%)
ASA 3	10 (34.5%)
ASA 3E	5 (17.2%)
ASA 4	2 (6.9%)
ASA 4E	2 (6.9%)
Difficult Intubation	2 (6.9%)
Extubation Time After Reversal (Median (IRQ))	14.50 (5-25.25)
Operation Characteristics	
Op Time (Median (IRQ)) mins	133 (43-178.50)
Vasopressors Required	4 (13.8%)
Hydromorphone Required	4 (13.8%)
Fentanyl (Median (IQR)) mcg	175 (45-250)
Morphine (Median (IQR)) mg	17.50 (4.50-25.0)
Crystalloid	800 (400-1250)
Colloid Given	5 (17.2%)
EBL	8 (0-100)
Blood Products Given	5 (17.2%)
Reason for Reintubation	
Mucus Plug	3 (10.3%)
Poor Respiratory Effort	6 (20.7%)
Cuff Leak	3 (10.3%)
Bleeding	4 (13.8%)
Laryngospasm or Bronchospasm	5 (17.2%)
Unintended Extubation	3 (10.3%)
Other	5 (17.2%)
Reintubation Characteristics	
Awake Reintubation	5 (17.2%)
Operating Room	28 (96.6%)

Predictors of reintubation

When controlling for operative time and case complexity via estimated blood loss, reintubation was predicted by high-dose opioids Odds ratio (OR) 9.467 95%CI 1.895-47.254 p = 0.006). Prolonged operative time, EBL, BMI, extubation time after reversal guided by the train of four monitoring, or age was not independently associated with UPR (Table 2).

**Table 2:** Predictors of Reintubation Table 2: Multivarate regression analysis depicting independent predictors of reintubation. High-Dose Opioids = morphine > 75 percentile or fentanyl > 75 percentile or hydromorphone Received; Prolonged/Increased.= >75 percentile

	OR (95%CI)	P value
Prolonged Operative Time	0.481 (0.092-2.514)	0.386
Increased EBL	0.733 (0.152-3.534)	0.699
Increased BMI	1.230 (0.253-5.986)	0.798
Prolonged Extubation time after reversal	5.850 (0.634-54.014)	0.119
High Dose Opioids	9.467 (1.897-47.254)	0.006
Age	0.995 (0.974-1.018)	0.686

## Discussion

Our single-center cohort study found an independent modifiable risk factor for UPR after general anesthesia: increased dose of opioids, leading to poor respiratory effort and hypoventilation. Opioid-induced respiratory depression (OIRD) is considered a diagnosis of exclusion, commonly identified by hypoventilation and oxygen desaturation, prior to eventual cardiac and respiratory embarrassment [9,10]. Adverse events from OIRD include a mean of 5-day increased hospital length of stay (LOS), 15.8% risk of readmission, and mean hospital cost increase of $10,00010. Anesthetic-induced factors including prolonged neuromuscular blockade and high opioid use have been described in the literature as leading to UPR [11,12]. Upper airway patency is controlled by muscle tone originating from the genioglossus and tensor palitini muscles. Perioperative administration of opioids reduces the ability of the aforementioned muscles to enable airway dilatation, leading to airway collapse, atelectasis, desaturation, and respiratory failure [13].

There are a number of additional factors that have been implicated in increased UPR rates, including patient-centric, operative, and anesthetic-related factors. Based upon our analysis, otolaryngology surgery was implicated in the risk for UPR, likely due to postoperative laryngeal edema, and potential hemorrhage at the surgical site. Rujirojindakul et al. identified head and neck surgery as an independent risk factor for UPR [14]. In a similar study by Chen et al., age>65, head-neck or thoracic surgery, American Society of Anesthesiology 3 classification, and high intravenous fluid administration were associated with the risk for UPR. These findings suggest that extubation should be cautiously undertaken and potentially even delayed in head and neck surgery until the risk of laryngeal edema has abated, warranting open communication between the surgical and anesthesiology services to avoid UPR. A study by Eikermann et al. found that patients were still vulnerable to developmental postoperative upper airway obstruction following recovery of the train of four ratios even in non-airway surgery [15]. Neurosurgical and dental procedures were also common culprits for UPR in our cohort, likely due to altered sensorium, involvement of lower cranial nerves, and procedural intervention in proximity to the airway, respectively. 

Patients experiencing UPR are more likely to suffer adverse outcomes. Ramachandran et al. reviewed over 220,000 elective, non-cardiac cases from the American College of Surgeons National Surgical Quality Improvement (NSQIP) Database, finding UPR was an independent risk factor for 30-day mortality [16]. Elmer et al. found that reintubated patients more commonly experienced perioperative complications including hypotension, hypoxia, and aspiration. (Elmer) UPR leads to a significant increase in healthcare costs, by increasing intensive care unit length of stay (ICU LOS), pneumonia rates, blood transfusion requirements, and an almost 50% increase in mortality risk [2,12,17]. A study by Menon et al. found that the mean hospital direct cost was $70,600 in patients who failed extubation versus $33,700 in those who did not [18]. In the early postoperative period, reintubation most commonly occurs within 0 to two hours after extubation and is less common after 24 hours [12,19]. The time to reintubation after extubation failure, defined as within 24 hours postoperatively in our study, has been described as an independent predictor of hospital mortality. UPR has also been shown to be a predictor of in-hospital mortality and discharge to a nursing home [20].

Higher mortality risk in patients with extubation failure and reintubation has multiple potential etiologies. The act of reintubation in an emergent situation can lead to adverse events due to underlying co-morbidities or complications from reintubation alone, with an incidence of death approximated at around 2% within 30 minutes of the procedure [21-23]. In a retrospective cross-sectional study by Zhang et al. investigating risk factors associated with reintubations in children undergoing foreign body removal body using flexible bronchoscopy, mortality associated with reintubation was up to 29% [24]. Data from observational studies reveal that 40 to 90% of patients show evidence of laryngeal damage or edema during laryngoscopy for reintubation [25-27]. Reintubated patients were also more likely to undergo ≥3 intubation attempts [28]. Furthermore, Thille et al. found that reintubated patients were more likely to incur aspiration, dental trauma, and esophageal intubation [29]. Torres et al. found that 47% of reintubated patients developed nosocomial pneumonia, particularly those with tracheobronchial colonization by pathogenic microorganisms, as compared to 10% of matched control patients [30].

In patients most vulnerable to UPR, risk stratification, anesthesia provider awareness, and education is mandatory to improve postoperative outcomes. The decision to extubate a patient undergoing head and neck surgery should be made judiciously in concert with surgical colleagues to avoid OIRD. Concerted minimization of opioid dosage and careful consideration of extubation criteria in high-risk patients will be instrumental in reducing this rare but devastating complication.

As a retrospective case-control study, there may be potential confounders (residual motor blockade, inadequate reversal of muscle relaxants) between the case and control groups leading to causal associations with unplanned reintubation. In addition, there are limitations to the granular data that can be ascertained from the electronic medical record prior to reintubation. Our study did not evaluate hospital LOS or total hospital cost, which are other important variables that warrant further study.

## Conclusions

It is imperative for anesthesia practitioners to be mindful of opioid-sparing techniques in high-risk patients. The ability to accurately identify patients at risk for extubation failure is essential in reducing patient morbidity and improving patient safety. Identification of patients at the highest risk for UPR will help guide providers in medical optimization, appropriate selection of intraoperative analgesics, and cautious extubation criteria to ensure patient safety. Prospective, multicenter trials with larger sample sizes are essential to validate UPR risk factors.
